# Segmentation, Detection, and Tracking of Stem Cell Image by Digital Twins and Lightweight Deep Learning

**DOI:** 10.1155/2022/6003293

**Published:** 2022-04-05

**Authors:** XiangXi Du, MuYun Liu, YanHua Sun

**Affiliations:** ^1^School of Mechanical Engineering, Xi'an Jiao Tong University, Xi'an City 710049, China; ^2^Shenzhen Cellauto Automation Co., Ltd., Shenzhen, China; ^3^School of Mechanical Engineering, Xi'an Jiao Tong University, Xi'an City 710049, China

## Abstract

The current work aims to strengthen the research of segmentation, detection, and tracking methods of stem cell image in the fields of regenerative medicine and tissue damage restoration. Firstly, based on the relevant theories of stem cell image segmentation, digital twins (DTs), and lightweight deep learning, a new phase contrast microscope is introduced through the research of optical microscope. Secondly, the results of DTs method and phase contrast imaging principle are compared in stem cell image segmentation and detection. Finally, a lightweight deep learning model is introduced in the segmentation and tracking of stem cell image to observe the gray value and mean value before and after stem cell image movement and stem cell division. The results show that phase contrast microscope can increase the phase contrast and amplitude difference of stem cell image and solve the problem of stem cell image segmentation to a certain extent. The detection results of DTs method are compared with phase contrast imaging principle. It indicates that not only can DTs method make the image contour more accurate and clearer, but also its accuracy, recall, and F1 score are 0.038, 0.024, and 0.043 higher than those of the phase contrast imaging method. The lightweight deep learning model is applied to the segmentation and tracking of stem cell image. It is found that the gray value and mean value of stem cell image before and after movement and stem cell division do not change significantly. Hence, the application of DTs and lightweight deep learning methods in the segmentation, detection, and tracking of stem cell image has great reference significance for the development of biology and medicine.

## 1. Introduction

The number of stem cell images is increasing rapidly; the traditional research methods and detection methods are not applicable and cannot meet the needs of production and life. In this case, developing new technologies and means is the requirement of the times [1, 2]. Digital twins (DTs) in stem cell image segmentation, detection, and tracking research can improve the work efficiency and avoid the loss caused by human errors [3]. American biologist Peter (2020) proposed a new method for stem cell image segmentation and detection. Its principle is to view the stem cell image as an undulating hill. The elevation of the hill represents the gray value of the image. When the elevation of the hill is falling and rising, the depression formed is a dividing line by which stem cells can be segmented by this dividing line [4]. Other scholars also commented on this method. They believe that although this method is simple and easy to operate, excessive segmentation appears in practical operation. Li (2021) used DTs and phase contrast imaging to analyze the phase contrast cell images by microscopes and proposed a segmentation method for restoring cell images according to the microscope imaging principle, which can remove the halo in the stem cell image and accurately segment the cells [5]. Jaccard (2020) used the maximum entropy and deep learning (DL) to realize the final segmentation, tracking, and detection by given different beam brightness in the area with high density of stem cells. In addition, he divided the stem cells images into two different types and applied different processing methods to different types of images [6]. Yang (2020) found that the phase difference imaging method can segment stem cell images more accurately, but it cannot process a large number of stem cell images because its processing logic process reduces the segmentation efficiency [7]. Topman (2021) used the stem cell activity trajectory marking and convolution neural network (CNN) model to draw the location, brightness, and division stage of each stem cell. Although the results obtained are accurate, it needs more time and the division and running tracks of stem cells are uncertain [8].

To sum up, based on DTs technology, at first, phase and amplitude of traditional microscope and phase contrast microscope are compared and analyzed. The most adaptive microscope is selected and compared with DTs technology. Next, the CNN model is used to study the stem cell image tracking. Present work aims to provide reference for further in-depth analysis of stem cells.

## 2. Theories and Methods

### 2.1. Construction of DTs Model Based on Image Analysis of Stem Cells

A stem cell is a type of cell that can produce other cells which are able to develop into any kind of cell in the body, so it is also known as the origin of cells. Stem cells are cells with unlimited or immortal self-renewal capacity, which can produce at least one type, highly differentiated progeny cells, so they are called “universal cells” in the medical profession [9–11]. According to the classification of developmental stages, stem cells can be divided into embryonic stem cells and adult stem cells [12–14].

**Figure 1:**
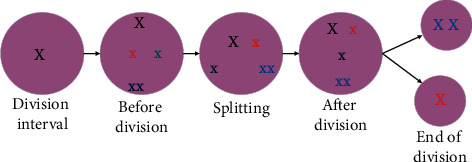
Specific process of cell division (x represents a cell).

Cell division mainly involves the division of the maternal cell, which divides into two daughter cells. Cell division is generally divided into nuclear and cytoplasmic division [15–17]. The maternal cells transmit genetic material to offspring mainly through nuclear division. Stem cells proliferate through mitosis. The cell division cycle is generally divided into interdivisional period and divisional period, and the whole cell division process is divided into prophase, metaphase, anaphase [18, 19].  Figure 1 demonstrates the specific process of cell division.

**Figure 2:**
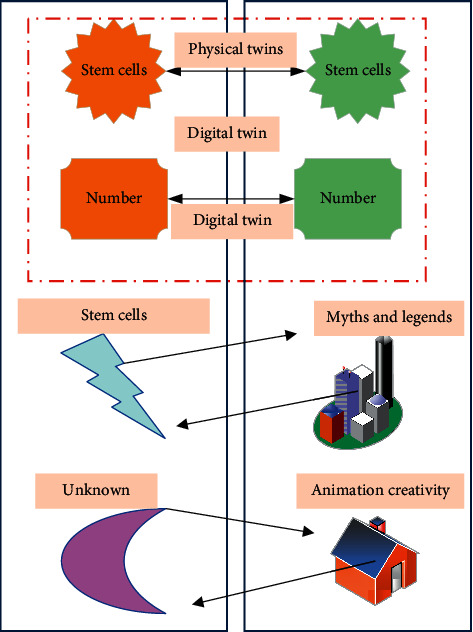
Research model of DTs.

DTs are one of the important models for detecting the segmentation results of stem cell images. They integrate the physical model, sensor update, operation history, and other data into a multidisciplinary, multiphysical quantity, multiscale, and multiprobability simulation process and complete mapping in virtual space, presenting the life cycle of corresponding physical equipment [20–22]. The DT model includes the offline model, the online model, and the model in the postprocessing and hypothesis verification stage [23, 24]. The process of stem cell image segmentation based on DTs is shown in Figure 2.

### 2.2. Research Methods

**Figure 3:**
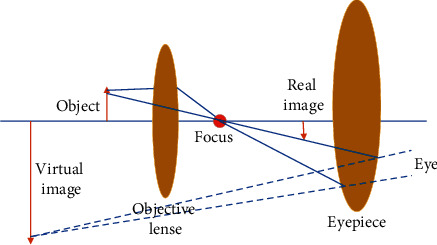
Principle of optical microscope imaging.

1. Comparative analysis method: Comparative analysis method refers to carrying out the multiparty comparison on two or more research objects to explore the similarities and differences between them and to analyze and learn from good methods, specifically for the present work, to accurately segment and detect stem cell images [2, 25, 26].
2. Literature review: Due to the writing needs of present work, at first, data query is carried out through the channels of China National Knowledge Infrastructure (CNKI), Google Academics, and Wanfang Data, where there are many original articles from columnists and related Internet information. Many journals and books related to the articles, such as “Hematopoietic Stem Cell Transplant Professional Standard Data Set,” “Mesenchymal Stem Cell Foundation and Clinical Second Edition,” and “Stem Cell Biology Foundation,” are read in the school library. Through the collection and summary of this series of data, a favorable theoretical basis is provided for the research ideas and methods of the present work [27–29].
3. Phase contrast imaging method: In real life, the most used image observation tool is the microscope.  Figure 3 signifies its specific imaging principle.

**Figure 4:**
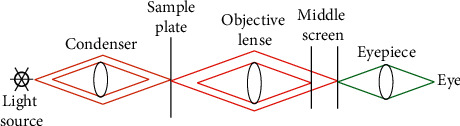
Specific imaging principle of phase contrast microscope.

Figure 3 reveals that the microscope uses the magnification imaging principle of the convex lens to magnify the objects that human beings cannot directly observe with eyes in life, so that the objects can be clearly distinguished by human eyes. The optical microscope has to perform imaging twice. According to the relevant knowledge of physics, the object after the first imaging is enlarged and inverted. According to the knowledge of optics, the object after the second imaging is shaped in an enlarged, upright virtual line. The object of present work is the stem cell. If the stem cell is not stained in advance, then it will be transparent, neither able to be observed by the naked eye, nor identified by microscope. Therefore, another phase contrast microscope is introduced, which is very different from the optical microscope.  Figure 4 illustrates its specific imaging principle.

**Figure 5:**
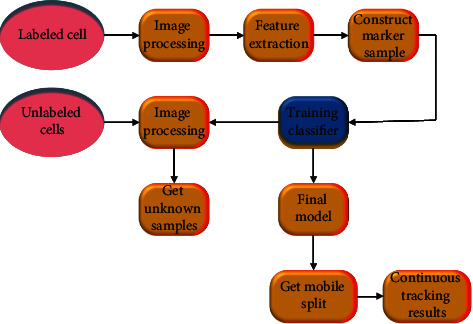
Steps of tracking stem cells images under CNN model.

By comparing Figures 3 and4, it can be found that a light source fence and a specimen plate are added to the phase contrast microscope. Different from the imaging principle of the optical microscope, the increased light source fence can gather the incoming beams to form a clearer light source than before. The effect of the specimen plate will move the light formed at the light source fence and then avoid the occurrence of the abovementioned situation that the phase contrast and amplitude difference are too close. (4) CNN model: it is a popular model in recent years. Due to its unique function, it plays an important role in stem cell image tracking.  Figure 5 shows its specific operation steps.

Figure 5 indicates that a classification idea is added to the CNN model, which plays an important role in the whole process of tracking stem cells. If there is a dislocation in the image labeling stage of stem cells, then there will be an error in the classification of CNN.

**Figure 6:**
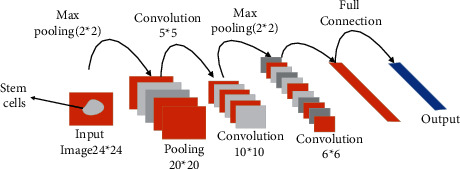
Stem cell image tracking based on CNN.

The size of the samples should be expanded because it is small. The performance of the CNN model is shown in Figure 6.

Figure 6 shows the CNN model modifies the corresponding output layer and adds two different output layers to the original one. This change can make the final result of stem cell division similar to the contour of original stem cells and improve the accuracy before and after stem cell segmentation.

### 2.3. Related Evaluation Index

Due to the particularity of segmentation and detection of stem cell images, the previous traditional classification accuracy cannot be used for the current evaluation. Therefore, F1-score and Area Under Curve (AUC) are introduced here. In the beginning, the confusion matrix is introduced, as shown in Table 1.

In Table 1, when the model predicts the true positive case BP as positive, its true positive label should also be positive; when the model predicts the true negative case EN as negative, its true positive label should also be negative; when the model predicts the false positive case EP as positive, its true positive label should also be negative; when the model predicts the false negative case BN as negative, its true positive label should also be positive.

*F1* score is an important indicator used to measure the effect of binary classification model, which is often seen in Statistics. It can be seen as a harmonic average of model accuracy and recall [30, 31]. It verifies between [0, 1]. According to the confusion matrix in Table 1, the corresponding expression can be obtained, as shown in

(1) $$\begin{array}{c} \text{Acc}=\frac{\text{BP}}{\text{BP}+\text{EP}}, \end{array}$$

(2) $$\begin{array}{c} \text{Rec}=\frac{\text{BP}}{\text{BP}+\text{BN}}, \end{array}$$

(3) $$\begin{array}{c} F_{1}=\frac{2\cdot \text{ACC}\cdot \text{Rec}}{\text{Acc}+\text{Rec}}. \end{array}$$

In equations (1)–(3), Acc represents the accuracy, and Rec refers to the recall rate. Equation (4) denotes the calculation of fast fitness.

(4) $$\begin{array}{c} \text{confluency}=\frac{\text{BP}+\text{EN}}{\text{BP}+\text{EN}+\text{BN}+\text{EP}}. \end{array}$$

The same signals in (4) have the same meaning with them in equations (1)–(3).

AUC is an important indicator of the quality of a binary classification model, which accords to the size of the area under the Receiver Operating Characteristic (ROC) curve. It represents the probability that the positive example is in front of the negative example [32, 33]. Its maximum value is 1 and the minimum value is 0.  Table 2 demonstrates its measurement standard.

The closer the AUC value is to 1, the better the classification effect can be achieved. The closer the AUC value is to 0, the worse the classification results will be obtained, which is not as good as the random guess method people usually use.

Equation (5) signifies the expression of AUC.

(5) $$\begin{array}{c} \text{AUC}=\frac{\sum_{i\in \text{positiveClass}}{\text{rank}}_{i}-C\left(1+C\right)/2}{C\cdot D}. \end{array}$$

In (5), C means the number of positive samples, and *D* represents the number of negative samples.

In the process of phase contrast microscope imaging, when the parallel light reaches the sample plate, its phase and amplitude are the same. Equation (6) specifies the specific calculation method.

(6) $$\begin{array}{c} \mu \left(x\right)=De^{\alpha ℑ}. \end{array}$$

In (6), *D* represents the amplitude, *ℑ* means the phase, *α* stands for the imaginary unit, and *e* refers to the angle of the parallel light. Parallel light will form two kinds of light waves when passing through the sample; one is the circumference wave and the other is the diffraction wave. Equations (7) and (8) denote the specific calculation methods.

(7) $$\begin{array}{c} \mu_{c}\left(x\right)=\mu \left(x\right)=De^{\alpha ℑ}. \end{array}$$

In (7), *μ*_*c*_(*x*) represents the circumference wave, and other signals have the same meaning as above. It can be seen that the calculation method of the surrounding wave is consistent with that of the amplitude.

(8) $$\begin{array}{c} \mu_{a}\left(x\right)=\varepsilon_{c}De^{\alpha \left(ℑ-f\left(x\right)\right)}. \end{array}$$

In (8), *ε*_*c*_ represents the amplitude attenuation coefficient, *f*(*x*) represents the phase shift, and the other signals have the same meanings as equations (9) and (10) illustrate the calculation methods of the surrounding wave and diffraction wave, under the idea that the phase *ℑ* before the light wave reaches the sample plate equals 0.

(9) $$\begin{array}{c} \mu_{c}\left(x\right)=\mu \left(x\right)=De^{\alpha 0}, \end{array}$$

(10) $$\begin{array}{c} \mu_{a}\left(x\right)=\varepsilon_{c}De^{-\alpha f\left(x\right)}. \end{array}$$

Signals in (9) and (10) have the same meanings as above. The effect of the phase plate can be equivalent to the band-pass filter. For the surrounding wave S, the phase plate can weaken the amplitude and make the phase advance a quarter of the wavelength. Equation (11) signifies the calculation method of the transmittance function.

(11) $$\begin{array}{c} P_{c}\left(t\right)=\varepsilon_{p}e^{\alpha 2/\pi }=\alpha \varepsilon_{p}. \end{array}$$

In (11), *P*_*c*_(*t*) represents the transmittance function, and signals in (9) and (10) have the same meanings as above.

When the diffraction wave passes through the phase plate, there will be some light penetrating the ring. Equation (12) displays the calculation method of its transmittance function.

(12) $$\begin{array}{c} P_{a}\left(t\right)=1+\left(\alpha \varepsilon_{p}-1\right). \end{array}$$

In (12), *P*_*a*_(*t*) represents another transmittance function, whose signals have the same meanings as above.

The consistency of the results needs to be tested to see how the stem cell image is segmented. The distance is adopted by many measurement methods of consistency as the index, among which the most important measurements methods are Euclidean distance, Minkowski distance, Manhattan distance, and Chebyshev distance [34–36].

(1) Euclidean distance
Euclidean distance is the actual distance between two points in the space, which is usual in daily lives. Equation (13) illustrates the calculation of it when adopted in the multidimension space.
  (13) $$\begin{array}{c} d_{pq}={\sqrt{\sum_{k-1}^{n}\left(d_{pk}-x_{qk}\right)}}^{2}. \end{array}$$
In (13), *d* represents the distance, *pq* denotes two points in the space, *n* refers to a multidimension space, *k* means the *k*_th_ point in the space, and *x* stands for the point formed by *pq*.
(2) Minkowski distance
Actually, Minkowski distance refers to a special form of Euclidean distance, which can also be written as Minkowski norm [37], whose calculation is shown in
  (14) $$\begin{array}{c} d\left(A,B\right)={\left(\sum_{i=1}^{n}\left|a_{i}-b_{i}\right|\right)}^{\frac{1}{p}}. \end{array}$$
 In (14), *A*, *B* represent two points in the space, *i* means the *i*_th_ point in the space, and *a*, *b* denotes the point formed by *A*, *B*. |*a*_*i*_ − *b*_*i*_| accords the distance of *i*_th_ point between *a*, *b* and *p* equals the times.
(3) Manhattan distance
 Manhattan distance expresses the sum of the definite wheelbases of two random points in coordinate system, whose calculation is shown in the following equation:

(15) $$\begin{array}{c} d\left(i,j\right)=\left|x_{1}-x_{2}\right|+\left|y_{1}-y_{2}\right|. \end{array}$$

In (15), *x*_1_ and *x*_2_ represent two points on the horizontal axis, separately, *y*_1_ and *y*_2_ denote two points on the longitudinal, respectively, *i* is the point formed by *x*_1_ and *y*_1_, and *j* refers to the point formed by *x*_2_ and *y*_2_. |*x*_1_ − *x*_2_| equals to the distance between *x*_1_ and *x*_2_, |*y*_1_ − *y*_2_| stands for the distance between *y*_1_ and *y*_2_, and the other signals have the same meaning as above.

## 3. Application Results and Analysis of DTs Based Detection and Tracking on Stem Cell Image Segmentation

### 3.1. Results Analysis of Detection on Stem Cell Images Segmentation

#### 3.1.1. Analysis of Stem Cell Image Segmentation Based on Phase Contrast Imaging Principle

**Figure 7:**
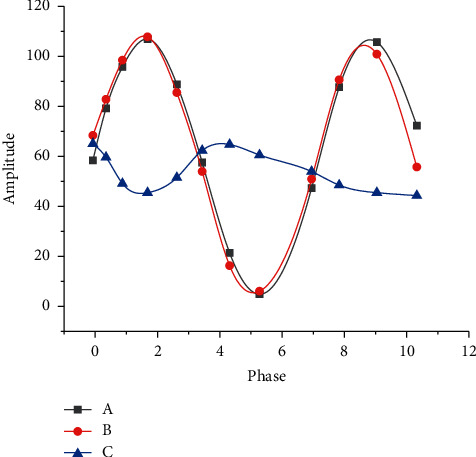
Relationship between phase and amplitude of the optical microscope.

Figure 7 reveals the relationship between phase and amplitude of the optical microscope.

In Figure 7, (a) represents the surrounding light whose beam did not pass through the sample plate, B refers to the very weak diffraction light, whose beam passes through the sample plate, and C is the beam formed by the superposition of the surrounding light and the diffraction light. Figure 6 suggests that the distance between C light and A light is very close, which indicates that their phase contrast and amplitude differences are very small, which brings difficulties to the image segmentation of stem cells.

**Figure 8:**
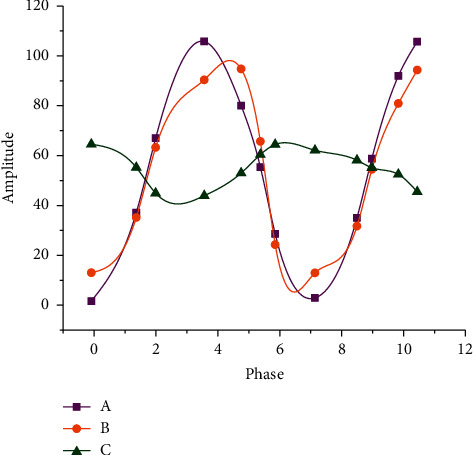
Relationship between the phase and amplitude of the phase contrast microscope.

Figure 8 unmasks the relationship between the phase and amplitude of the phase contrast microscope.

Figure 8 indicates that the phase and amplitude of the phase contrast microscope have changed after being added with the light source fence and the specimen plate, and the distance between the C light and the A light formed by the superposition becomes larger, which indicates that the difference between the phase and the amplitude becomes larger, which solves the difficulty of stem cell image segmentation to a certain extent.

#### 3.1.2. Analysis of Stem Cell Image Segmentation Based on DTs

**Figure 9:**
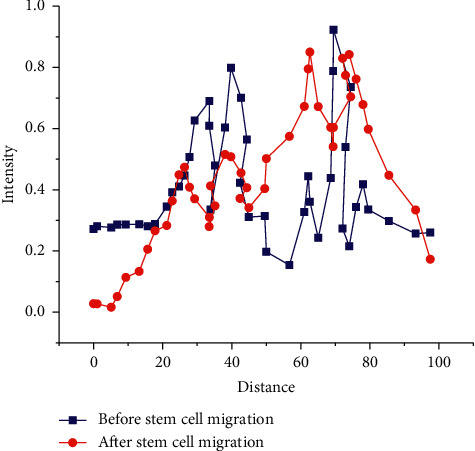
Intensity distribution of stem cell image before and after processing.

Before detecting the results of stem cell, segmentation should be carried out on the stem cells firstly, and the intensity distribution of stem cell images should be observed. However, there are some problems in the distribution of unprocessed images at the level of stem cell segmentation.  Figure 9 presents the specific intensity changes before and after processing.

Figure 9 displays that the intensity of stem cell images presents a trend of fluctuation in the distribution, and there will be overlaps between 0–25 mm and 55–75 mm, which brings some difficulties to the study of segmentation and detection of stem cell image. To change this situation, it is necessary to process the images before segmentation, to reduce the overlap between cells. The distribution of stem cell images after processing is as shown in the red lines in Figure 9. The fluctuation amplitude in Figure 8 is smaller than that before processing, and the difference between the background pixel and the target pixel is clearer.

**Figure 10:**
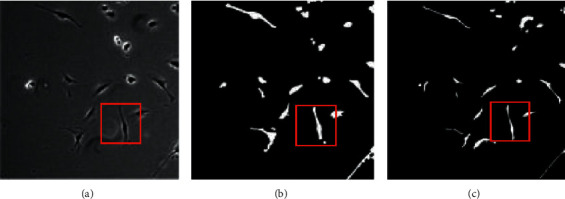
Comparison of two segmentation methods of stem cell image with the original image; (a) the original stem cell image; (b) the segmentation result of stem cell image under the phase contrast imaging method; (c) the segmentation result of stem cell image under DTs.

Present work mainly uses the DTs method and phase contrast imaging principle to segment the image of stem cells. To compare the difference between the two methods comprehensively and accurately, the segmentation results of the two methods are compared, whose specific results are shown in Figure 10.

From the comparison in Figure 10, it can be found that the contour of the stem cell image under the DTs is clearer and more obvious, and the overall imaging effect is also more realistic. However, the accuracy of stem cell image under the phase contrast imaging method is not high, which can be judged from the cell in Figure 9. The cells in the phase contrast imaging method are larger than those in the original image, and their contours are also very blurred. Besides, in the images of cells under strong light, there will be exposure. It can be seen that, in this comparison, the final effect of DTs is better. Additionally, the accuracy, recall, and F1 scores of the two methods are also compared, and the specific results are shown in Table 3.

The results are obtained by using the consistency test equation, as shown in Table 3. Table 3 indicates that the accuracy of phase contrast imaging method is 0.924, the accuracy of DTs method is 0.962, the recall rate of phase contrast imaging is 0.861, the recall rate of DTs method is 0.885, the F1 score of phase contrast imaging method is 0.902, and the F2 score of DTs method is 0.945. Hence, whether it is accuracy, recall, or F1 scores of DTs that are relatively high, in the comparison of segmentation of stem cell image, the results of DTs are the best.

### 3.2. Analysis on the Tracking and Detection of Stem Cell Images

**Figure 11:**
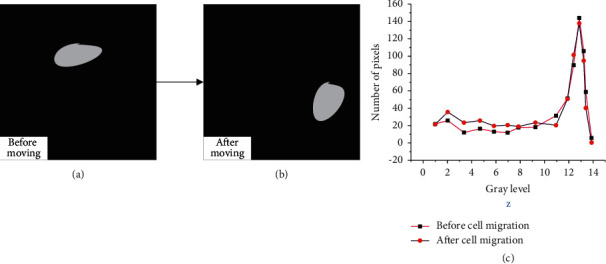
Comparison of the variances of stem cell before and after movements: (a) before the movements of stem cells; (b) after the movements of stem cells; (c) the changing trend of the grade degree before and after the movements of stem cells.

The movement of stem cells will cause impact on the results.  Figure 11 illustrates the variance before and after movements.

**Figure 12:**
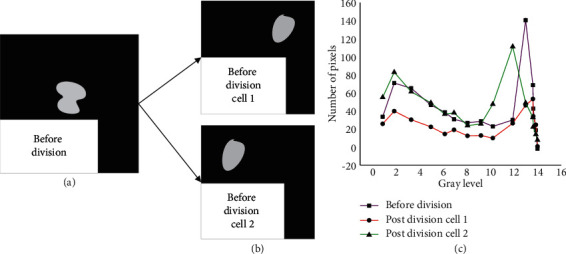
Comparison of stem cells before and after division: (a) stem cell before division; (b) cell 1 after division; (c) cell 2 after division.

Figure 11 shows that the changes of stem cells before and after the movement are not significant. On the whole, the changing trend presents a small fluctuation in the early stage, a sharp rise in the middle stage, and a sudden decline in the final stage. The changing trend before and after division is also discussed, and the results are shown in Figure 12.

**Figure 13:**
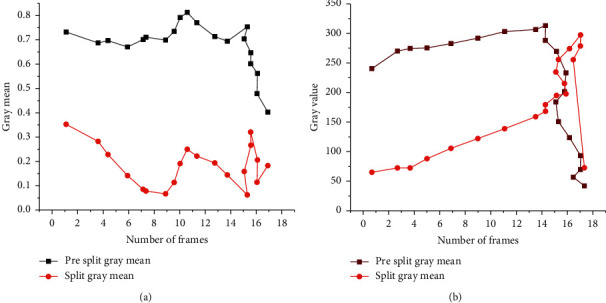
Variance of mean and gray degree of cells: (a) the variance of the mean of gray degree; (b) variance of gray degree.

Figure 11 implies that when the stem cells are divided into two daughter cells, the overall change is roughly the same, showing a trend of rising first and then falling. The only difference is that cell 2 after division is significantly different from the stem cell before division. Under the same gray degree, the number of single cells decreases, and the maximum pixel number also decreases. Besides, to better show the change of stem cell mean, a dot-line diagram is drawn, whose specific results are shown in Figure 13.

Figure 13 implies that, compared with before the division of stem cells, the changing trend of gray degree declines to 0.4–0.51 and keeps being stable within 0.42–0.52, during 20–60 fps. When the stem cell just divides, there is no obvious difference between the mean of gray degree before the division and after division; the sum of gray degree after division shows up with a continuously surging trend, compared with that before the division. Then it shows up with a descending trend when the gray degree after the division has achieved the maximum of that before the division. Besides, when the stem cells divide, similar to the changes of gray mean value before and after the division, the difference between the total gray value before and after the division is not obvious.

## 4. Discussion

According to the relevant theories of DTs, the segmentation, detection, and tracking methods of stem cell images are discussed. It is found that the accuracy rate, recall rate, and F1-score of stem cell images are the best, which provides research ideas and methods for stem cell image segmentation, detection, and tracking. In the United States, there are many theoretical methods for stem cell image analysis. The specific procedure is that the relevant information of stem cells is quickly and accurately extracted, segmented, and detected. Ansari (2020) used phase difference imaging to segment stem cells and made great achievements. However, because this kind of method needs to model the collected image and real image according to the phase difference imaging principle, it takes a long time and cannot apply to large-scale cells [38]. Yin (2021) used the level set method to realize cell tracking and determine the cell trajectory in the time dimension. In each frame, the number of cells, their location, their boundary, their region, and their states are calculated to detect cell division. The detection results of division events depend on the performance of cell tracking. Because it is difficult to track cells accurately, these methods have certain limitations [39]. As the basis of tracking and recognition, cell image segmentation is very important. At present, the segmentation effect of the phase difference stem cell images with low convergence is the best. But the effect is poor under high convergence. Therefore, how to improve the cell segmentation performance needs further research.

## 5. Conclusions

After DTs are applied to stem cell image segmentation, detection, and tracking, the following conclusions are drawn: the contour of stem cell images is clearer, and the images become more realistic after the phase difference imaging principle is compared with DTs in stem cell image segmentation and detection. In addition, the accuracy rate, recall rate, and F1-score of stem cell images under DTs are 0.962, 0.885, and 0.945, respectively, while those under the phase difference imaging principle are 0.924, 0.861, and 0.902, respectively. This proves that DTs have a better performance in stem cell image segmentation. After the stem cell image is tracked with CNN, it is found that the mean value, gray value, and image change trend before and after the movement and division do not change much.

Due to the limited energy, there are still limitations in data acquisition, resulting in some deviations in the analysis. In addition, the economic investment of the application of DTs to stem cell image segmentation, detection, and tracking is not discussed, and the subsequent benefit evaluation will be carried out according to the specific situations. This study brings some beneficial empirical results for stem cell image segmentation and tracking.

## Figures and Tables

**Table 1:** Confusion matrix.

Content	Real category	
Guess category	BP	EP
	BN	EN

**Table 2:** AUC measurement standard.

Range	Significance
AUC = 1	Perfect classification
1 > AUC > 0.5	Good predictive power
AUC = 0.5	No predictive power
0.5 > AUC > 0	It is better to guess at random

**Table 3:** Comparison of phase contrast imaging and DTs.

Type	Phase contrast imaging method	DTs principle
Accuracy	0.924	0.962
Recall	0.861	0.885
F_1_ fraction	0.902	0.945
Average processing time	13.245	2.314

## Data Availability

The research data used to support the findings of this study are included within the article.
